# Risk factors for angiotensin converting enzyme inhibitor angioedema in a South African population

**DOI:** 10.3389/falgy.2025.1664354

**Published:** 2025-10-20

**Authors:** Cascia Day, Lovemore Mapahla, Melissa Ribeiro, Mimi Deetlefs, Cathryn McDougall, Adelein Engelbrecht, Erika Jones, Sarah Pedretti, Jonny Peter

**Affiliations:** 1 Division of Allergology and Clinical Immunology, Department of Medicine, Faculty of Health Sciences, University of Cape Town, Cape Town, South Africa; 2 Allergy and Immunology Unit, University of Cape Town Lung Institute, Cape Town, South Africa; 3 The Division of Epidemiology and Biostatistics, Department of Global Health, Stellenbosch University, Stellenbosch, South Africa; 4 The Modelling and Simulation Hub, Africa, Department of Statistical Sciences, University of Cape Town, Cape Town, South Africa; 5 District 6 Day Hospital, Western Cape, Cape Town, South Africa; 6 The Division of Nephrology and Hypertension, Department of Medicine, Faculty of Health Sciences, University of Cape Town, Cape Town, South Africa

**Keywords:** angioedema, angiotensin converting enzyme inhibitor, ACEI angioedema, Africa, drug latency

## Abstract

**Introduction:**

Angiotensin converting enzyme inhibitors (ACEI) have proven mortality and morbidity benefit in hypertension, ischemic heart disease, heart failure, and renal disease and are among the most prescribed medications globally. ACEI angioedema (AE-ACEI) is a potentially life-threatening adverse drug reaction that is reported to occur more frequently in African American populations. However, the clinical profile of AE-ACEI is poorly characterized in diverse African populations.

**Methods:**

A case-controlled cohort study with enrolment of AE-ACEI cases and drug-tolerant controls in Cape Town, South Africa. Univariable and multivariable analysis was performed. Controls were defined as patients tolerating ACEI for a minimum of two years. Cases were defined as patients who had angioedema while using an ACEI, patients with a history of angioedema while not on an ACEI were excluded. Cases and controls were recruited from the same demographic areas, including both hospitals and clinics. Information regarding demographics and clinical history was captured via both in person interviews and folder review.

**Results:**

A total of 237 AE-ACEI cases, and 466 ACEI tolerant controls were enrolled from seven sites in Cape Town. Features of IgE-mediated immediate drug hypersensitivity were present in 24 cases, which excluded them from analysis. The median age was 58 years (IQR: 47; 67) and 57% were female. AE-ACEI cases more frequently had Black genetic ancestry compared to controls [53% (81/154), vs. 29% (146/407), *p* < 0.001]. AE-ACEI occurred within 30 days of initiating ACEI therapy in only 31.1% (70/225), with median treatment time to AE-ACEI of 6.9 years (IQR: 2.9; 13). The ACEI tolerant controls were using ACEI for median 9.5 years (IQR: 5; 15.5). All AE-ACEI cases developed swelling above the shoulders, involving the lips and tongue in 72% (165/213) and 50% (107/213) cases respectively. Hospitalisation for AE-ACEI was required in 82% (175/213), however only two patients were intubated, and there were no mortalities. In multivariable analysis traditional risk factors of age, gender, immunosuppression and atopy did not differ between cases and controls. Black genetic ancestry [aOR 15.4 (95% CI 2.94–283), *p* value = 0.01] and calcium channel blocker use [aOR 1.77 (95% CI 1.17–2.72), *p* value = 0.008] were significant risk factors for developing AE-ACEI. Cardiac failure, chronic kidney disease, and statin use reduced the risk of AE-ACEI in this model.

**Conclusion:**

In this South African population, Black genetic ancestry and calcium channel blocker use were the major risk factors for AE-ACEI. The majority of AE-ACEI occurred after several years of treatment, with most cases involving the lip and/or tongue. Long-term follow-up, genetic, and further mechanistic studies are warranted in additional diverse African populations.

## Introduction

Angiotensin converting enzyme inhibitors (ACEI) are a class of antihypertension medications that inhibit the Angiotensin Converting Enzyme (ACE; also known as kinase II). ACE is responsible for the conversion of angiotensin I to angiotensin II, as well as degradation of the vasoactive peptide bradykinin. ACEI are one of the most widely used medications globally as a result of their affordability, and many large studies have demonstrated their efficacy in the treatment of hypertension (1), cardiac failure (2, 3), ischemic heart disease (4), and chronic renal disease (5). ACEI also show improved efficacy, when compared to angiotensin receptor blockers (ARBs), in preventing hypertension related cardiovascular events, and all-cause mortality (6). Baptiste et al. found that when comparing ACEI to ARBs in hypertensive patients that in the Black African cohort ARB use was associated with a higher risk of cardiovascular related death when compared to ACEI [HR 1.2 (95% CI: 1.02–1.4)]. The two major adverse drug reactions with ACEI are cough and angioedema.

Angioedema is defined as localised swelling in subcutaneous and submucosal tissues. The frequency of AE-ACEI varies from 0.2% to 1% (7), but ubiquitous use of these medications means that AE-ACEI is the most common single cause of angioedema globally, including in South Africa (8–11). AE-ACEI most commonly affects the head and neck (12–14), with mortality rates reported as high as 11%, and intubation rates at 22% internationally (9, 12, 15). Several studies have investigated epidemiological and clinical risk factors for AE-ACEI (see Table 1) with consistent identified risk factors including: African American ancestry (with a 3–5 times increased AE-ACEI angioedema frequency) (10, 13, 16–18, 20, 23, 26, 40), female gender (17, 20, 21), older age (12, 16, 20–22), use of immunosuppression (25, 29, 30), and seasonal allergies (16, 21, 36). Additional identified risk factors (with some conflicting findings) include a variety of concomitant medications (18, 20–22, 25, 30, 41, 42), current smoking status (16, 23–25), and obesity (16, 22). The recent initiation of ACEI and first 30 days of treatment with ACEI have previously been identified as the highest risk period for AE-ACEI (17, 18, 26, 29, 37–39). Most of these epidemiological studies of AE-ACEI are from High Income Countries (HICs) and predominantly European populations, despite the reported increased risk in African Americans. Thus, the aim of this study was to examine the clinical profile and assess risk factors for AE-ACEI in an African setting.

**Table 1:** Risk factors for ACEI angioedema from existing published studies.

Intrinsic patient risk factors	Environmental factors
African race as risk factor (10, 13, 16–18) and not as risk factor (12, 19) described	Habits
Age greater than or equal to 65 years (12, 16, 20–22)	Smoking (16, 23–25)
Female gender (17, 20, 21)	Medication
Male gender (22)	Use of Calcium Channel blockers (21, 22, 24)
Increased BMI (16, 22)	Use of antihistamines (21)
Comorbid illness	Use of NSAIDs (20, 22, 26)
C1 inhibitor deficiency or defect (27, 28)	Use of immunosuppressants (25, 29, 30)
History of allergic rhinitis (16, 21)	Use of diuretic (18, 22)
Seasonal allergies (10, 21, 25)	Use of systemic corticosteroids (21)
DM increased risk (12, 16) and protection described (10, 21)	Higher ACEI doses (18)
Hypertension (12, 16)	Use of statins (22)
Rheumatoid Arthritis (21)	Use of anti-diabetic drugs (22)
COPD (21)	Other
History of drug induced rash (10)	Trauma (30–33)
CKD (25)	Recent hospitalisation (18)
Hyperlipidaemia (16)	Pollen (34, 35)
Autoimmune disease (16)	Spring season (36)
Rheumatoid arthritis (21)	
Known drug allergy (20)	
Solid organ transplant (25, 30)	
Malignancy (22)	
Anaemia (22)	
Atopic dermatitis (16)	
Other factors	
Highest rate of AE-ACEI within 30 days of starting ACEI (17, 18, 26, 29, 37–39)	
Recent reinitiation of ACEI (18)	
Unilateral angioedema (12)	
Absence of urticaria or itch (12)	
Angioedema of the lips (12)	
Polypharmacy (22, 24)	
No history of other ACEI use (23)	
ACEI cough (23)	

Yellow cells indicate conflicting data.

ACEI, angiotensin converting enzyme inhibitor; AE-ACEI, angiotensin converting enzyme inhibitor angioedema; BMI, body mass index; CKD, Chronic Kidney Disease; COPD, Chronic Obstructive Pulmonary Disease; DM, diabetes mellitus; NSAIDS, non-steroidal anti-inflammatory drugs.

## Methods

This is a case control study comparing patients who tolerate ACEI vs. patients who have developed AE-ACEI. We aim to describe this cohort of patients and identify significant risk factors for AE-ACEI in this community. AE-ACEI cases were defined as participants who developed angioedema while on an ACEI, with no history of angioedema while not using an ACEI. ACEI tolerant controls were defined as participants who had safely tolerated an ACEI for at least two years, with no history of angioedema. In this study, AE-ACEI cases were identified both prospectively (through referral) and retrospectively (through folder review). Cases and matched controls were recruited from seven sites: District 6 Clinic, Victoria Wynberg Hospital, Mitchell's Plain District Hospital, Heideveld Emergency Centre, Green Point Clinic, The University of Cape Town Lung Institute Allergy and Immunology Clinic, and Groote Schuur Hospital (GSH) (allergy division, emergency unit, hypertension clinic, and general medical wards). All the above facilities are primary or secondary level centers that refer to GSH and the demographics of this cohort match the demographics of the Western Cape Province (see Supplement for more information about each facility, a detailed description of recruitment at each site as well as consort diagrams, Supplementary Figures 1, 2). The Western Cape Provincial Health Data Centre (PHDC) team assisted with accessing HECTIS admission data (43). All cases and controls were interviewed in person, and all data was assessed by an Allergist/Allergy Medical Officer. Information regarding angioedema history, medical history, and demographics was collected. This study is part of the Angioedema Biomarkers in Africa project, which has been approved by the University of Cape Town Human Research Ethics Committee (HREC 057/2020).

We acknowledge that neither self-reported race nor Fitpatrick skin tone accurately captures genetic ancestry. Therefore, we have decided to group patients based on our available genetic ancestry data into White ancestry, Admixed ancestry, and Black ancestry based on location and grouping in the Principle Component Analysis plot (Supplementary Figure 3) from our previous genome wide association study in this cohort. We did not have genotype data for 118/679 (17.4%) of patients.

Proportions and frequencies were used to describe categorical variables. Normally distributed variables, tested using the Shapiro Wilk test of normality, were described using mean and standard deviation or else median and interquartile range was used. The mean difference test in continuous variables that were normally distributed between groups was done using the analysis of variance (ANOVA) or else the Kruskal Wallis test was used. Proportion difference test for all categorical variables that had at least 5 observations across all groups was done using the Chi square test of independence, or else the Fisher's exact test was applied. All statistical tests were done at 5% level of significance, and Stata 15.1 software was used for the analyses (44). Multivariable analysis was performed for this cohort with AE-ACEI as the outcome. Patients were stratified as “hospitalised” (including patients in general medical wards, intensive care or high care units, and the emergency center) or “not hospitalised” (patients from outpatient departments.). The following covariates were included: age, gender, skin tone, atopy, Human immunodeficiency virus (HIV), hypertension, elevated cholesterol, cardiac failure, and chronic kidney disease. Key selected medications included: immunosuppression, NSAIDs, Statins, and Calcium channel blockers. Cardiac disease was removed from the model as there was evidence of collinearity with cardiac failure. Multicollinearity was assessed using The Variance Inflation Factor (VIF) (Supplementary Table 2), and all results were less than 5. Adjusted odds ratios (aOR) with respective 95% confidence intervals (CI) were reported. There was minimal missing data in chosen co-variates (see Supplementary Table 6). We performed Multivariable analysis for this cohort with AE-ACEI within 30 days of initiating treatment as the outcome, but no covariates reached significance (Supplementary Table 3).

## Results

A total of 237 AE-ACEI cases and 466 ACEI tolerant controls were enrolled. Between June 2021 and December 2024, 49 cases of acute AE-ACEI were referred to the GSH Allergy team, while 188 participants with a history of prior AE-ACEI were retrospectively enrolled via folder review and interview. In the AE-ACEI angioedema cases, 24 participants were excluded, as they had evidence of immediate drug hypersensitivity (urticaria *n* = 10; pruritis *n* = 13; anaphylaxis *n* = 1) as determined by two allergists (Supplementary Figures 1, 2). Most of our cohort were treated with the ACEI enalapril [99.7%, (699/701)]. In the ACEI tolerant controls the duration on an ACEI at the time of enrollment was median 9.5 years (IQR: 5; 15.5 years).

In the overall cohort, the median age was 58 years (IQR: 47; 67), 57.5% were female, and 94.8% (532/561) were classified as Admixed or Black ancestry (see Table 2). The cases and controls were similar in terms of age and gender, but significantly more AE-ACEI cases were classified as Black ancestry when compared to controls [53% (81/154), vs. 29%% (119/407), *p* < 0.001]. With regards to comorbid illness, 97.5% of patients had hypertension. HIV was more prevalent in the AE-ACEI cases [16.9% (36/213) vs. 11.4% (53/466), *p* = 0.043] but immunosuppressive treatment did not differ between cases and controls. Compared to cases the controls had significantly higher rates of hypercholesterolaemia [51.9% (242/466) vs. 39.0% (83/213), *p* = 0.001], cardiac disease [21.9% (102/466) vs. 12.2% (26/213), *p* = 0.006], or had previously tested positive for COVID-19 [16.3% (76/466) vs. 12.2% (26/213), *p* = 0.001]. Atopy was reported in 32.3% (69/213) of cases and 27.6% (126/466) of controls but the results did not reach significance (*p* = 0.201). The cases had significantly higher rates of asthma [42.0% (29/69) vs. 20.9% (27/129), *p* = 0.001], while atopic dermatitis was more common in the controls [31% (40/129) vs. 8.6% (8/69), *p* = 0.023]. Calcium channel blockers use was significantly greater in the cases [135/213 (63%) vs. 232/466 (50%), *p* < 0.001] while simvastatin use was significantly more common in the controls [267/466 (57%) vs. 88/213 (41%), *p* < 0.001]. In multivariable analysis Black ancestry (aOR 15.3; 95% CI 2.94–283, *p* = 0.01), calcium channel blocker usage (aOR 1.77; 95% CI: 1.17–2.72, *p* = 0.008) were significant risk factors for developing AE-ACEI (Table 3). Chronic kidney disease, cardiac failure, and statin use were protective in this model.

**Table 2:** Univariate comparison of self-reported clinical variables between ACEI angioedema cases and ACEI tolerant controls and prevalence of previously reported risk factors.

Variable	All, *n* = 679	ACEI angioedema cases, *n* = 213	Controls, *n* = 466	*P* values
Age, years med (IQR)	58 (47;67)	57 (47;68)	59 (46;67)	0.7
Female, n(%)	390 (57.5)	132 (62.0)	258 (55.4)	0.092
Ancestry from genotype, n(%), *n* = 561				
White	29 (5.2)	1 (0.6)	28 (6.9)	**<0.001**
Admixed	332 (59)	72 (47)	260 (64)	
Black	200 (36)	81 (53)	119 (29)	
Genotype not available	118 (17.4)	59 (27.6)	59 (12.6)	
Location of recruitment				
Out patient department	425 (62.6)	122 (57.3)	304 (65.2)	**0**.**0281**
Emergency room	221 (32.5)	81 (38)	140 (30)	
General inpatient	31 (4.6)	8 (3.8)	23 (4.9)	
High care or intensive care unit	2 (0.3)	2 (0.9)	0 (0)	
Comorbidities^1^, n(%)				
Hypertension	662 (97.5)	210 (98.6)	452 (97)	0.20
Hypercholesterolemia	325 (47.9)	83 (39)	242 (51.9)	**0**.**002**
Diabetes	237 (34.9)	67 (31.5)	170 (36.5)	0.202
Atopy	195 (28.7)	69 (32.3)	126 (27.6)	0.201
Cardiac disease^2^	128 (18.9)	27 (12.2)	101 (21.9)	**0**.**006**
Cardiac failure	119 (17.5)	20 (9.4)	99 (21.2)	**<0**.**001**
HIV	89 (13.2)	36 (16.9)	53 (11.4)	**0**.**043**
Vascular disease^3^	53 (8)	15 (7)	38 (8.2)	0.615
Chronic kidney disease	51 (7.5)	10 (4.7)	41 (8.8)	0.060
Prevalence of previously reported risk factors for AE-ACEI, n(%)				
Fitz Patrick V-VI skin tone	254 (38.3)	108 (50.7)	146 (31.3)	**<0**.**001**
Over 65 years old	219 (32.3)	63 (29.6)	156 (33.5)	0.313
Allergic rhinitis	117 (17.2)	37 (17.4)	80 (17.2)	0.948
On immunosuppression	34 (5)	8 (3.8)	26 (5.6)	0.312
NSAID use	162 (23.9)	43 (20)	119 (26)	0.129
Diuretic use	465 (68)	154 (72.0)	311 (67.0)	0.148
Statin use	355 (52.3)	88 (41.0)	267 (57.0)	**<0**.**001**
Calcium channel blocker use	367 (54.1)	135 (63)	232 (50.0)	**0**.**001**
Oral steroids	7 (1.1)	4 (1.9)	3 (0.6)	0.089
Oral antihistamines	68 (10.6)	19 (8.9)	49 (10.5)	0.900
>3 medications (polypharmacy)	565 (83.2)	181 (85.0)	384 (82.4)	0.405
Increased stress		47 (22.1)		
Acute illness		20 (9.4)		
New chronic illness		18 (8.5)		
Use of over the counter medications		16 (17.0)		

ACEI, angiotensin converting enzyme inhibitor; AR, allergic rhinitis; CKD, chronic kidney disease; HIV, Human immunodeficiency virus; IQR, interquartile range, LABA, long acting beta agonist, NSAID, non-steroidal anti-inflammatory; SABA, short acting beta agonist.

Bold *p* values indicate statistical significance.

1 Only comorbidities >5% included in table. Others include: abnormal uterine bleeding *n* = 1; acute renal injury *n* = 1; allergic conjunctivitis *n* = 1; anxiety *n* = 3; anemia *n* = 1; arthritis *n* = 60; atopic dermatitis *n* = 7; B12 deficiency *n* = 1; bronchitis *n* = 1; cancer *n* = 10; cellulitis *n* = 1; chronic constipation *n* = 3; chronic gastroenteritis *n* = 1; chronic pain *n* = 10; clubfoot *n* = 1; COPD *n* = 9; Crohn's disease *n* = 1; current TB *n* = 6; depression *n* = 7; dermatitis *n* = 2; dyslipidemia *n* = 3; epilepsy *n* = 14; fibromyalgia *n* = 1; glaucoma *n* = 2; GORD *n* = 36; gout *n* = 36; headache *n* = 2; hernia *n* = 3; hip replacement *n* = 1; IgA disease *n* = 2; jaundice *n* = 1; meningitis *n* = 1; mixed connective tissue disease *n* = 1; muscle spasms *n* = 1; myasthenia gravis *n* = 1; obesity *n* = 2; overactive bladder *n* = 1; Penicillin allergy *n* = 1; peptic ulcer disease *n* = 3; polio *n* = 1; porphyria *n* = 2; post herpetic neuralgia *n* = 1; PPE *n* = 1; previous TB *n* = 80; prolapsed lumbar disc *n* = 1; prostate disease *n* = 2; psoriasis *n* = 2; pulmonary embolism *n* = 2; retinopathy *n* = 1; rheumatic hear disease *n* = 1; rheumatoid arthritis *n* = 8; schistosomiasis *n* = 1; schizoaffective disorder *n* = 1; silicosis *n* = 1; sinusitis *n* = 1; systemic lupus erythramatosis *n* = 10; spinal injury *n* = 1; gastrointestinal infection *n* = 1; tinea *n* = 2; thyroid disease *n* = 19; trisomy 21 *n* = 1; ulcerative colitis *n* = 1, urinary tract infection *n* = 1.

2 Cardiac disease: valvular heart disease and ischaemic heart disease.

3 Vascular disease: stroke and peripheral vascular disease.

**Table 3:** Unadjusted and adjusted analysis of clinical risk factors for ACEI angioedema.

Characteristic	Unadjusted			Adjusted		
	OR	95% CI OR	*p*-value	aOR	95% CI aOR	*p*-value
Age	1.00	0.99–1.01	0.70	1.01	1.01–1	0.11
Male	0.75	0.54–1.05	0.092	0.95	0.62–1.45	0.8
Admixed ancestry	7.75	1.61–139	0.046	6.16	1.22–112	0.081
Black ancestry	19.10	3.94–343	0.004	15.30	2.94–283	**0**.**01**
Atopy	1.23	0.87–1.75	0.2	1.44	0.92–2.25	0.11
HIV	1.60	1.01–2.52	0.045	1.08	0.58–1.98	0.8
Hypertension	2.17	0.70–9.48	0.20	0.3	0.05–1.87	0.2
Elevated cholesterol	0.60	0.43–0.83	0.002	1.44	0.78–2.70	0.20
Cardiac Failure	0.39	0.23–0.63	<0.001	0.43	0.22–0.82	**0**.**014**
Chronic Kidney Disease	0.51	0.24–1.00	0.064	0.24	0.05–0.72	**0**.**024**
Immunosuppressive drug use	0.66	0.28–1.42	0.3	0.94	0.29–2.62	>0.90
NSAID use	0.74	0.49–1.09	0.13	1.23	0.69–2.15	0.50
Statin use	0.52	0.38–0.73	<0.001	0.36	0.19–0.65	**<0**.**001**
Calcium channel blocker use	1.75	1.25–2.44	0.001	1.77	1.17–2.72	**0**.**008**
Hospitalised	1.39	0.99–1.93	0.053	1.66	1.07–2.58	**0**.**024**

aOR, adjusted odds ratio; HIV, human immunodeficiency virus; NSAID, non-steroidal anti-inflammatory drug; OR, odds ratio.

Bold *p* values indicate statistical significance.

In our cohort the duration of ACEI treatment before developing angioedema median of 6.9 years (IQR: 2.9; 13.0 years) with 31.5% (67/213) of patients developing angioedema within 30 days of ACEI initiation. The median duration of AE-ACEI was 48 h (IQR: 24; 72 h). Multivariable analysis for developing AE-ACEI within 30 days of initiating treatment found no significant covariates (Supplementary Table 3).

**Figure 1:**
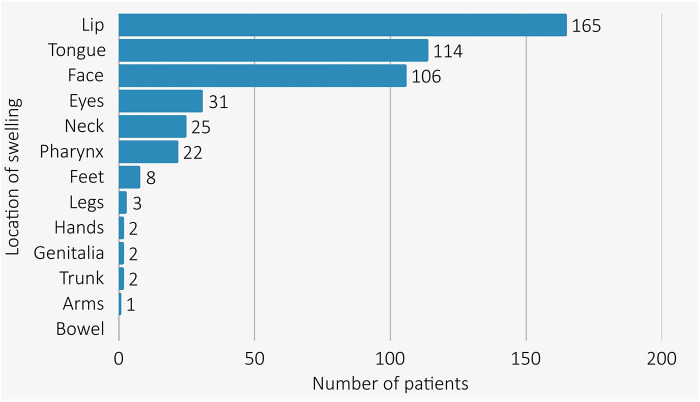
Bar graph describing distribution of AE-ACEI location in cases.

All AE-ACEI cases had swelling above the shoulders (see Figure 1) with the lip [72.3% (154/213)] and tongue [50.2% (107/213)] being the most common sites of swelling. Overall, 22 (10.3%) patients developed pharyngeal angioedema (10/22 with concomitant tongue and lip swelling), with two patients requiring intubation for airway protection (one emergency orotracheal intubation, and one emergency cricothyroidotomy both after reinitiation of an ACEI in patients with known AE-ACEI). There were no angioedema related deaths. Hospitalisation was required in 82.2% (175/213) of AE-ACEI cases. Information about treatment of AE-ACEI was available for 41.8% of cases (89/213). Therapy with fresh frozen plasma was given in 11 cases (11/89, 12.4%) while 73% (65/89) were treated with antihistamines, 64% (57/89) with corticosteroids, and 12.4% (12/89) required adrenaline for airway protection. One patient received Icatibant peri-intubation after discussion with our Angioedema Hotline.

## Discussion

AE-ACEI is a common and potentially life-threatening complication of ACEI use, and the global rate of acute ACEI requiring care in emergency centers is rising (45, 46). To date there are no available tests or biomarkers to predict which patients are at risk of developing AE-ACEI. Currently, clinical risk factors remain the best clues to identify at-risk groups—however, these risk factors may be population-specific with limited data from diverse African countries. This large South African cohort of AE-ACEI and drug-tolerant controls were examined for traditional and novel risk factors. AE-ACEI cases more commonly had Black genetic ancestry, a higher prevalence of HIV, and lower rates of concomitant metabolic and non-hypertensive cardiovascular diseases. Notably, the majority of AE-ACEI occurred following prolonged periods of treatment, and there was increased occurrence during the spring/summer seasons. Interestingly, we also found that all patients had angioedema of the face and neck. Despite high rates of hospitalisation there were low rates of intubation (0.9%) for airway protection, and no deaths. This is contradictory to international data where 22% (37) to 32% (13) of patients with AE-ACEI required intubation, and 11% demised (37). Unlike international groups, we did not find that older age, female gender, allergic rhinitis, or immunosuppressive therapy were associated with AE-ACEI. However, similar to international data, we found that calcium channel blockers were a significant risk factor (21, 22, 24).

In 1996, Brown et al. (18) were the first to describe an increased risk for AE-ACEI in African Americans with a relative risk of 4.5 compared to white Americans. They also found that African patients were more likely to have severe hypertensive disease as they required higher doses of ACEI, as well as having more severe AE-ACEI with higher rates of hospitalisation and intubation for airway protection. Later studies in the USA (10, 13, 16, 17, 20, 23, 41) and a systematic review (40) found that black and Hispanic patients had a significantly higher risk of AE-ACEI compared with patients with paler skin tone (41). However, other authors from the USA have found that African race were not associated with AE-ACEI (12, 19). Studies completed in Sweden, the United Kingdom, and Thailand either did not describe racial demographics (21), or had no patients with darker skin tones (24, 39) (see Supplementary Table 5 for a summary of these studies). Our data from an African Middle Income Country (MIC) setting does find a significantly higher prevalence of Black genetic ancestry in AE-ACEI cases with a high aOR of 15.3 (*p* = 0.01 (Table 3). This result aligns with African American data and suggests that examining African populations for genetic risk factors for AE-ACEI is warranted. We have recently published a GWAS from this cohort and we replicated findings for the single nucleotide polymorphisms (SNP) rs500766 on chromosome 10 (previously linked to AE-ACEI). We also found SNPs located close to the genes *PRKCQ* (protein kinase C theta), *RAD51B* (RAD51 Paralog B), and *RIMS1* (regulating synaptic membrane exocytosis 1), which have previously been linked with drug-induced angioedema. Additionally, SNPs near the *CSMD1* (CUB and sushi multiple domains 1) gene, which has been linked to ACEI cough (47), were identified. We have highlighted that further work across diverse African populations is justified, as given African genomic diversity extrapolation to all darker skinned populations would be flawed (48).

AE-ACEI can occur immediately upon drug exposure, or years into therapy, and its occurrence cannot be predicted. Some authors have found that AE-ACEI is most common within the first 30 days of starting treatment, reporting that 48.6–53.0% of AE-ACEI cases occur within that window (17, 18, 39), while others have found much lower AE-ACEI rates of 10.2% within the first month of treatment (15, 39, 41). Our cohort had a long drug latency, with only 31.1% developing AE-ACEI within the first 30 days of drug initiation, which is within range of global rates. In AE-ACEI this prolonged drug latency raises the question of whether there is an additional factor/second hit event in the late presenters which i) either effects other enzymes that metabolise bradykinin, such as neprilysin, or aminopeptidase P, ii) may represent another factor that leads to increased flux through the bradykinin pathway, potentially even at the tissue-level, or iii) represents the onset of a chronic urticaria/angioedema variant independent of AE-ACEI. A recent publication by Bocquet et al. found that, on review of reported AE-ACEI angioedema cases that almost 50% of patients were excluded, and that these patients likely had mast cell mediated angioedema rather than AE-ACEI (49).

All of our AE-ACEI cases presented with swelling above the shoulders, most commonly affecting the lips and tongue, aligning with global findings (14, 36). Previous studies have identified tongue swelling in AE-ACEI as a marker associated with poor clinical outcomes and increased likelihood of intensive care admissions (13, 15). Other forms of bradykinin mediated angioedema—such as hereditary angioedema and acquired angioedema—can affect multiple body sites, including the oropharynx, abdominal viscera, limbs, and genitalia (50). If AE-ACEI is also bradykinin mediated, one would expect it to involve a broader range of anatomical sites. The density of mast cells in tissue is higher in the face than other anatomical areas (51); so a possible explanation is that ACEI treatment is unmasking mast cell mediated angioedema (i.e., ACEI are acting as a cofactor). This is supported by recent findings of high rates of mast cell mediated angioedema in patients who developed recurrent angioedema after exposure to ACEI. Douillard et al. found that 41% of patients with a suspected ACEI/ARB angioedema still had recurrent angioedema without urticaria more than 6 months after stopping the drug, indicating a diagnosis of mast cell mediated angioedema rather than bradykinin mediated angioedema in these patients (52). These findings are important and underline the need for future investigations into the pathomechanisms of AE-ACEI, and potentially cross-talk between mast-cells and kallikrein-kinin pathways.

This study is the largest study to date describing AE-ACEI in an African MIC. Our limitations include prospective case finding, which was reliant on referrals from local sites; as well as retrospective case finding which is dependent on existing ICD10 coding and local databases. We encountered difficulties in contacting retrospective cases to schedule in-person interviews because of a highly migrant population, and incorrect or outdated contact details. Our cohort had higher rates of hypertension and dyslipidaemia than reported in South Africa (53), which likely reflects our recruitment from hospitals and local clinics, rather than the general population. However, ACEI are most commonly prescribed for hypertension, so high hypertension rates (and associated metabolic conditions) in this cohort are not an unexpected finding. The case-controlled nature of the study cannot exclude potential for unintentional selection bias; however, cases and controls were recruited from the same hospitals and drainage populations. It is also important to note that as this study is nested in the government funded state sector, almost all our cohort were using Enalapril, which is the ACEI on government tender. We did have some variables with high rates of missing data—especially with regards to treatment of AE-ACEI—as participants either did not know or could not recall the treatment that was received at the time of the event.

In summary, we have shown that Black genetic ancestry, calcium channel blocker use, and HIV co-infection are important risk factors for AE-ACEI—and that previously identified risk factors including older age, female sex, and allergic rhinitis were not risk factors in this cohort. We have also identified a long drug latency compared to international cohorts. These findings highlight the need for further epidemiological and clinical studies on AE-ACEI in diverse ethnic backgrounds and LMIC settings to provide a true global clinical understanding of AE-ACEI.

## Data Availability

The raw data supporting the conclusions of this article will be made available by the authors, without undue reservation.

## References

[1] Williams GH. Converting-enzyme inhibitors in the treatment of hypertension. N Engl J Med. (1988) 319(23):1517–25. 10.1056/NEJM198812083192305 3054561

[2] Swedberg K Kjekshus J, CONSENSUS Trial Study Group. Effects of enalapril on mortality in severe congestive heart failure. Results of the Cooperative North Scandinavian Enalapril Survival Study (CONSENSUS). N Engl J Med. (1987) 316(23):1429–35. 10.1056/NEJM198706043162301 2883575

[3] Yusuf S Pitt B Davis CE Hood WB Cohn JN. Effect of enalapril on survival in patients with reduced left ventricular ejection fractions and congestive heart failure. N Engl J Med. (1991) 325(5):293–302. 10.1056/NEJM199108013250501 2057034

[4] TAIREA Study. Effect of ramipril on mortality and morbidity of survivors of acute myocardial infarction with clinical evidence of heart failure. The acute infarction ramipril efficacy (AIRE) study investigators. Lancet. (1993) 342(8875):821–8. 10.1016/0140-6736(93)92693-N 8104270

[5] Lewis EJ Hunsicker LG Bain RP Rohde RD. The effect of angiotensin-converting-enzyme inhibition on diabetic nephropathy. The collaborative study group. N Engl J Med. (1993) 329(20):1456–62. 10.1056/NEJM199311113292004 8413456

[6] Peresuodei TS Gill A Orji C Reghefaoui M Saavedra Palacios MS Nath TS. A comparative study of the safety and efficacy between angiotensin-converting enzyme inhibitors and angiotensin receptor blockers on the management of hypertension: a systematic review. Cureus. (2024) 16(2):e54311. 10.7759/cureus.54311 38496070 PMC10944326

[7] Kostis JB Packer M Black HR Schmieder R Henry D Levy E. Omapatrilat and enalapril in patients with hypertension: the omapatrilat cardiovascular treatment vs. Enalapril (OCTAVE) trial. Am J Hypertens. (2004) 17(2):103–11. 10.1016/j.amjhyper.2003.09.014 14751650

[8] Maurer M Magerl M. Differences and similarities in the mechanisms and clinical expression of bradykinin-mediated vs. Mast cell-mediated angioedema. Clin Rev Allergy Immunol. (2021) 61(1):40–9. 10.1007/s12016-021-08841-w 33534062 PMC8282544

[9] Day C van der Walt J Crombie K Hendrikse C Peter J. Acute angioedema in Cape Town emergency centres and a suggested algorithm to simplify and improve management. S Afr Med J. (2023) 113(8):51–7. 10.7196/SAMJ.2023.v113i8.717 37882115

[10] Kostis JB Kim HJ Rusnak J Casale T Kaplan A Corren J Incidence and characteristics of angioedema associated with enalapril. Arch Intern Med. (2005) 165(14):1637–42. 10.1001/archinte.165.14.1637 16043683

[11] Hoover T Lippmann M Grouzmann E Marceau F Herscu P. Angiotensin converting enzyme inhibitor induced angio-oedema: a review of the pathophysiology and risk factors. Clin Exp Allergy. (2010) 40(1):50–61. 10.1111/j.1365-2222.2009.03323.x 19659669

[12] Bluestein HM Hoover TA Banerji AS Camargo CA Jr Reshef A Herscu P. Angiotensin-converting enzyme inhibitor-induced angioedema in a community hospital emergency department. Ann Allergy Asthma Immunol. (2009) 103(6):502–7. 10.1016/S1081-1206(10)60267-0 20084844

[13] Chan NJ Soliman AM. Angiotensin converting enzyme inhibitor-related angioedema: onset, presentation, and management. Ann Otol Rhinol Laryngol. (2015) 124(2):89–96. 10.1177/0003489414543069 25059449

[14] Zingale LC Beltrami L Zanichelli A Maggioni L Pappalardo E Cicardi B Angioedema without urticaria: a large clinical survey. Can Med Assoc J. (2006) 175(9):1065–70. 10.1503/cmaj.060535 17060655 PMC1609157

[15] Agah R Bandi V Guntupalli KK. Angioedema: the role of ACE inhibitors and factors associated with poor clinical outcome. Intensive Care Med. (1997) 23(7):793–6. 10.1007/s001340050413 9290997

[16] Kamil RJ Jerschow E Loftus PA Tan M Fried MP Smith RV Case-control study evaluating competing risk factors for angioedema in a high-risk population. Laryngoscope. (2016) 126(8):1823–30. 10.1002/lary.25821 27426939 PMC4955864

[17] Reichman ME Wernecke M Graham DJ Liao J Yap J Chillarige Y Antihypertensive drug associated angioedema: effect modification by race/ethnicity. Pharmacoepidemiol Drug Saf. (2017) 26(10):1190–6. 10.1002/pds.4260 28722207

[18] Brown NJ Ray WA Snowden M Griffin MR. Black Americans have an increased rate of angiotensin converting enzyme inhibitor-associated angioedema. Clin Pharmacol Ther. (1996) 60(1):8–13. 10.1016/S0009-9236(96)90161-7 8689816

[19] Chiu AG Newkirk KA Davidson BJ Burningham AR Krowiak EJ Deeb ZE. Angiotensin-converting enzyme inhibitor-induced angioedema: a multicenter review and an algorithm for airway management. Ann Otol Rhinol Laryngol. (2001) 110(9):834–40. 10.1177/000348940111000906 11558759

[20] Garcia-Saucedo JC Trejo-Gutierrez JF Volcheck GW Park MA Gonzalez-Estrada A. Incidence and risk factors of angiotensin-converting enzyme inhibitor-induced angioedema: a large case-control study. Ann Allergy Asthma Immunol. (2021) 127(5):591–2. 10.1016/j.anai.2021.07.028 34363973

[21] Mahmoudpour SH Baranova EV Souverein PC Asselbergs FW de Boer A Maitland-van der Zee AH. Determinants of angiotensin-converting enzyme inhibitor (ACEI) intolerance and angioedema in the UK clinical practice research datalink. Br J Clin Pharmacol. (2016) 82(6):1647–59. 10.1111/bcp.13090 27524468 PMC5099558

[22] Stauber T Confino-Cohen R Goldberg A. Life-threatening angioedema induced by angiotensin-converting enzyme inhibitors: characteristics and risk factors. Am J Rhinol Allergy. (2014) 28(1):54–8. 10.2500/ajra.2014.28.3989 24717884

[23] Morimoto T Gandhi TK Fiskio JM Seger AC So JW Cook EF An evaluation of risk factors for adverse drug events associated with angiotensin-converting enzyme inhibitors. J Eval Clin Pract. (2004) 10(4):499–509. 10.1111/j.1365-2753.2003.00484.x 15482412

[24] Hallberg P Nagy J Karawajczyk M Nordang L Islander G Norling P Comparison of clinical factors between patients with angiotensin-converting enzyme inhibitor-induced angioedema and cough. Ann Pharmacother. (2017) 51(4):293–300. 10.1177/1060028016682251 27889699

[25] Byrd JB Woodard-Grice A Stone E Lucisano A Schaefer H Yu C Association of angiotensin-converting enzyme inhibitor-associated angioedema with transplant and immunosuppressant use. Allergy. (2010) 65(11):1381–7. 10.1111/j.1398-9995.2010.02398.x 20557296 PMC3305268

[26] Banerji A Clark S Blanda M LoVecchio F Snyder B Camargo CA Jr. Multicenter study of patients with angiotensin-converting enzyme inhibitor-induced angioedema who present to the emergency department. Ann Allergy Asthma Immunol. (2008) 100(4):327–32. 10.1016/S1081-1206(10)60594-7 18450117

[27] Cichon S Martin L Hennies HC Müller F Van Driessche K Karpushova A Increased activity of coagulation factor XII (hageman factor) causes hereditary angioedema type III. Am J Hum Genet. (2006) 79(6):1098–104. 10.1086/509899 17186468 PMC1698720

[28] Bowen T Cicardi M Bork K Zuraw B Frank M Ritchie B Hereditary angiodema: a current state-of-the-art review, VII: canadian Hungarian 2007 international consensus algorithm for the diagnosis, therapy, and management of hereditary angioedema. Ann Allergy Asthma Immunol. (2008) 100(1 Suppl 2):S30–40. 10.1016/S1081-1206(10)60584-4 18220150

[29] Byrd JB Touzin K Sile S Gainer JV Yu C Nadeau J Dipeptidyl peptidase IV in angiotensin-converting enzyme inhibitor associated angioedema. Hypertension. (2008) 51(1):141–7. 10.1161/HYPERTENSIONAHA.107.096552 18025295 PMC2749928

[30] Abbosh J Anderson JA Levine AB Kupin WL. Angiotensin converting enzyme inhibitor-induced angioedema more prevalent in transplant patients. Ann Allergy Asthma Immunol. (1999) 82(5):473–6. 10.1016/S1081-1206(10)62723-8 10353579

[31] Megerian CA Arnold JE Berger M. Angioedema: 5 years’ experience, with a review of the disorder’s presentation and treatment. Laryngoscope. (1992) 102(3):256–60. 10.1288/00005537-199203000-00005 1545652

[32] Vleeming W van Amsterdam JG Stricker BH de Wildt DJ. ACE inhibitor-induced angioedema. Incidence, prevention and management. Drug Saf. (1998) 18(3):171–88. 10.2165/00002018-199818030-00003 9530537

[33] Schiller PI Messmer SL Haefeli WE Schlienger RG Bircher AJ. Angiotensin-converting enzyme inhibitor-induced angioedema: late onset, irregular course, and potential role of triggers. Allergy. (1997) 52(4):432–5. 10.1111/j.1398-9995.1997.tb01024.x 9188926

[34] Wilson M Frohna W Trent G Sauter D. Evaluating for seasonal variation in angiotensin-converting enzyme inhibitor- and angiotensin receptor blocker-induced angioedema. Ann Allergy Asthma Immunol. (2014) 112(2):178–9. 10.1016/j.anai.2013.11.016 24468261

[35] Straka B Nian H Sloan C Byrd JB Woodard-Grice A Yu C Pollen count and presentation of angiotensin-converting enzyme inhibitor-associated angioedema. J Allergy Clin Immunol Pract. (2013) 1(5):468–73.e1–4. 10.1016/j.jaip.2013.05.003 24565618 PMC4042396

[36] Pfaue A Schuler PJ Mayer B Hoffmann TK Greve J Hahn J. Clinical features of angioedema induced by renin-angiotensin-aldosterone system inhibition: a retrospective analysis of 84 patients. J Community Hosp Intern Med Perspect. (2019) 9(6):453–9. 10.1080/20009666.2019.1698259 32002148 PMC6968333

[37] Slater EE Merrill DD Guess HA Roylance PJ Cooper WD Inman WH Clinical profile of angioedema associated with angiotensin converting-enzyme inhibition. JAMA. (1988) 260(7):967–70. 10.1001/jama.1988.03410070095035 2840522

[38] Hedner T Samuelsson O Lunde H Lindholm L Andrén L Wiholm BE. Angio-oedema in relation to treatment with angiotensin converting enzyme inhibitors. Br Med J. (1992) 304(6832):941–6. 10.1136/bmj.304.6832.941 1581715 PMC1882283

[39] Win TS Chaiyakunapruk N Suwankesawong W Dilokthornsakul P Nathisuwan S. Renin angiotensin system blockers-associated angioedema in the Thai population: analysis from Thai national pharmacovigilance database. Asian Pac J Allergy Immunol. (2015) 33(3):227–35. 10.12932/AP0556.33.3.2015 26342120

[40] McDowell SE Coleman JJ Ferner RE. Systematic review and meta-analysis of ethnic differences in risks of adverse reactions to drugs used in cardiovascular medicine. Br Med J. (2006) 332(7551):1177–81. 10.1136/bmj.38803.528113.55 16679330 PMC1463974

[41] Banerji A Blumenthal KG Lai KH Zhou L. Epidemiology of ACE inhibitor angioedema utilizing a large electronic health record. J Allergy Clin Immunol Pract. (2017) 5(3):744–9. 10.1016/j.jaip.2017.02.018 28377081 PMC5476207

[42] Hallberg P Persson M Axelsson T Cavalli M Norling P Johansson HE Genetic variants associated with angiotensin-converting enzyme inhibitor-induced cough: a genome-wide association study in a Swedish population. Pharmacogenomics. (2017) 18(3):201–13. 10.2217/pgs-2016-0184 28084903

[43] Boulle A Heekes A Tiffin N Smith M Mutemaringa T Zinyakatira N Data centre profile: the provincial health data centre of the Western Cape Province, South Africa. Int J Popul Data Sci. (2019) 4(2):1143. 10.23889/ijpds.v4i2.1143 32935043 PMC7482518

[44] StataCorp. Release Fifteen. College Station, TX: StataCorp LLC (2017).

[45] Cicardi M Suffritti C Perego F Caccia S. Novelties in the diagnosis and treatment of angioedema. J Investig Allergol Clin Immunol. (2016) 26(4):212–21; quiz two pages after page 21. 10.18176/jiaci.0087 27470642

[46] Smith A Ray M Jain N Zhang H Sebelik M. The burden of angioedema on United States emergency departments: 2006–2010. Laryngoscope. (2017) 127(4):828–34. 10.1002/lary.26336 27861934

[47] Mugo JW Day C Choudhury A Deetlefs M Freercks R Geraty S A GWAS of angiotensin-converting enzyme inhibitor-induced angioedema in a South African population. J Allergy Clin Immunol Glob. (2025) 4(3):100464. 10.1016/j.jacig.2025.100464 40290521 PMC12022653

[48] Peter JG Ntusi NAB Ntsekhe M. Are recommendations that favor other agents over angiotensin-converting enzyme inhibitors in Africans with hypertension justified? Circulation. (2024) 149(11):804–6. 10.1161/CIRCULATIONAHA.123.065887 38466787

[49] Bocquet A Marmion N Boccon-Gibod I Bouillet L. Angiotensin-converting enzyme inhibitor-induced angioedema: proposal for a diagnostic score. World Allergy Organ J. (2025) 18(3):101037. 10.1016/j.waojou.2025.101037 40151541 PMC11946868

[50] Day C Peter J. The South African ACARE centre approach to recurrent angioedema without urticaria. Curr Allergy Clin Immunol. (2023) 36(3):148–53.

[51] Weber A Knop J Maurer M. Pattern analysis of human cutaneous mast cell populations by total body surface mapping. Br J Dermatol. (2003) 148(2):224–8. 10.1046/j.1365-2133.2003.05090.x 12588371

[52] Douillard M Deheb Z Bozon A Raison-Peyron N Dereure O Moulis L Over diagnosis of bradykinin angioedema in patients treated with angiotensin-converting enzyme inhibitors or angiotensin II receptor blockers. World Allergy Organ J. (2023) 16(8):100809. 10.1016/j.waojou.2023.100809 37638360 PMC10458346

[53] Peer N Uthman OA Kengne A-P. Rising prevalence, and improved but suboptimal management, of hypertension in South Africa: a comparison of two national surveys. Glob Epidemiol. (2021) 3:100063. 10.1016/j.gloepi.2021.100063 37635713 PMC10445958

