# Supplementary material for: Risk factors for angiotensin converting enzyme inhibitor angioedema in a South African population
Source: Front Allergy. 2025 Oct 20;6:1664354. doi: 10.3389/falgy.2025.1664354 (PMC12580374; doi:10.3389/falgy.2025.1664354)
Supplement: Supplementary file 1 [file Datasheet1.docx]

**Supplementary material**

**Supplementary Table 1: Participants stratified by location**

| **Variable** | **All, n=679** | **ACEI-angioedema cases, n=213** | **Controls, n=466** |
| --- | --- | --- | --- |
| Sampling Location |  |  |  |
| Groote Schuur Hospital (telephonic, home visit) | 505 (74.4) | 160 (75.1) | 345 (74.0) |
| District 6 Clinic | 129 (19.0) | 11 (5.2) | 118 (25.3) |
| Victoria Wynberg Hospital | 6 (0.8) | 6 (2.8) | 0 |
| Mitchells Plain District Hospital and Heideveld Emergency Center | 8 (1.2) | 8 (3.8) | 0 |
| Green Point Clinic | 1 (0.1) | 1 (0.5) | 0 |
| UCT Lung Institute Allergy and Immunology Clinic | 31 (4.6) | 6 (2.8) | 25 (5.4) |

Facility descriptions

Participant screening and enrollment occurred at seven sites within the Cape Town Metro health district, which serves a population of approximately 3.3 million people. This includes one private clinic (UCT Lung Institute Allergy and Immunology Clinic), two primary health care clinics (District 6 Clinic, Green Point Clinic), two district/secondary hospitals (Mitchells Plain District Hospital and Victoria Wynberg Hospital), and finally one tertiary referral hospital (Groote Schuur Hospital).

Groote Schuur Hospital (GSH) (505 patients)

GSH opened in 1938, the hospital has a bed capacity of 893 and serves the entirety of the Western Cape Western Metropol. The hospital offers both tertiary-level services, receiving referrals from secondary-level hospitals (George Regional Hospital, Victoria Wynberg Hospital, New Somerset Hospital, and Mitchells Plain District Hospital) and secondary-level services. The patient population has high rates of communicable and non-communicable diseases. Attending clinical teams are consultant physician led teams usually consisting of medical interns, medical officers, and specialist registrars in internal medicine. Sub-specialist patient consultation is readily available. Full radiology and laboratory services are available 24 hours.

GSH recruitment flow:

1. Prospective AE-ACEI cases: (n=23)
   1. These were patients who were referred to the on call allergist between 2021-2025 with AE-ACEI. Patients who consented, who were within 48hours of the start of swelling, and were still swollen at the time of referral were included. If the patient was not swollen or if they were out of the 48hour time frame they were sampled as retrospective cases with genetic testing only.
2. Retrospective AE-ACEI cases: (n=137)
   1. These patients were identified by three methods:
      1. Screening all Emergency Center admissions using the HECTIS programme, with the ICD10 T78.3 (Angioedema)
      2. Screening all folders at the GSH Hypertension Clinic
      3. Referral of historical cases of AE-ACEI from other OPDs to the Allergist on call
3. ACEI tolerant controls: (n=345)
   1. These were defined as patients who had tolerated an ACEI for at least two years without AE-ACEI, with no history of other angioedema
      1. Screening all folders at the GSH Hypertension Clinic
      2. Screening of patients at the Allergy Clinic
      3. Screening of general hospital medical wards

Victoria Hospital, Wynberg (VHW) (6 patients)

VHW opened in 1889 has a bed capacity of 206. The hospital offers primary and secondary level services and serves the Southern Peninsula Health District, as well as Pollsmoor Prison. Attending clinical teams are consultant physician led teams usually consisting of medical interns, medical officers, and rotating specialist registrars. VWH does not have an obstetric or neonatal service. Patients are referred for sub-specialist care to GSH e.g. renal dialysis, prolonged ICU care. Radiology services include 24 hours-a-day X-rays and working hours CT and ultrasonography. All laboratory investigations are sent to the NHLS laboratory at GSH.

VWH recruitment flow:

1. Prospective AE-ACEI cases: (n=6)
   1. These were patients who were referred to the on call allergist between 2021-2025 with AE-ACEI. Patients who consented, who were within 48hours of the start of swelling, and were still swollen at the time of referral were included. If the patient was not swollen or if they were out of the 48hour time frame they were sampled as retrospective cases with genetic testing only.

Mitchells Plain District Hospital (MPDH), and Heideveld Emergency Center (HEC) (8 patients)

MPH was built in 2010 has a bed capacity of 200. The hospital offers both primary and secondary-level services. Attending clinical teams are consultant physician led teams usually consisting of medical interns, officers, and specialist registrars in internal medicine. Patients are referred for sub-specialist care to GSH e.g. renal dialysis and ICU/ high care unit (HCU). Radiology services include 24 hours-a-day X-rays and working hours ultrasonography and CT scanning. All laboratory investigations (barring histology) are sent to the accredited NHLS laboratory located on site. HEC is at the site of the old Jooste Hospital and is a standalone emergency facility linked to MPDH.

MPDH recruitment flow:

1. Prospective AE-ACEI cases: (n=8)
   1. These were patients who were referred to the on call allergist between 2021-2025 with AE-ACEI. Patients who consented, who were within 48hours of the start of swelling, and were still swollen at the time of referral were included. If the patient was not swollen or if they were out of the 48hour time frame they were sampled as retrospective cases with genetic testing only.
      1. HEC n=3
      2. MPDH n=5

District 6 Clinic

This clinic was opened in 2018 and operates in the Cape Town Central Health District of the Metro Region. Services are offered to a population of 70000 from Woodstock, Salt River, Vredehoek, Bo –Kaap, City Bowl, and other surrounding areas, as well as clients commuting into the CBD. The District Six CDC operates from 07:30 am to 16:00 from Monday to Friday. Services offered include: women’s health, family planning, antenatal care, termination of pregnancy (TOP); integrated chronic disease management consisting of non-communicable and communicable diseases; mental health services; antiretroviral service of over 5000 clients; minor surgical procedures; dietetics; chronic medicine collection; orthopaedic nursing outreach; wound care and dermatology; and pharmacy services.

D6 recruitment flow:

1. Prospective AE-ACEI cases: (n=3)
   1. These were patients who were referred to the on call allergist between 2021-2025 with AE-ACEI. Patients who consented, who were within 48hours of the start of swelling, and were still swollen at the time of referral were included. If the patient was not swollen or if they were out of the 48hour time frame they were sampled as retrospective cases with genetic testing only.
2. Retrospective AE-ACEI cases: (n=8)
   1. These patients were identified by identification and folder review of patients who were using Losartan at the pharmacy
   2. Referral of historical cases of AE-ACEI from D6 to the Allergist on call
3. ACEI tolerant controls: (n=118)
   1. These were defined as patients who had tolerated an ACEI for at least two years without AE-ACEI, with no history of other angioedema
      - 1. Screening all folders at the D6 Chronic Disease Unit
        2. Screening of patients who were prescribed an ACEI at the pharmacy

UCT Lung Institute Allergy and Immunology Clinic

A specialised Allergy and Immunology referral clinic in the private health sector and sees patients from around the world. This clinic is run by five Allergists (one Family Medicine Physician, two Paediatric Allergists, and two Physician Allergists) in addition to two Allergy Nurses. This clinic has full resuscitation facilities and is also linked to the onsite Clinical Trials unit.

UCT Lung Institute Allergy and Immunology Clinic recruitment flow:

1. Retrospective AE-ACEI cases: (n=6)
   1. Referral of historical cases of AE-ACEI from UCT Lung Institute Allergy and Immunology to the Allergist on call
2. ACEI tolerant controls: (n=25)
   1. Referral from UCT Lung Institute Allergy and Immunology to the study team

Green Point Clinic

This clinic operates in the Cape Town Central Health District of the Metro Region and offers basic medical services, primarily via nursing teams.

Green Point Clinic recruitment flow:

1. Prospective AE-ACEI cases: (n=1)

These were patients who were referred to the on call allergist between 2021-2025 with AE-ACEI. Patients who consented, who were within 48hours of the start of swelling, and were still swollen at the time of referral were included. If the patient was not swollen or if they were out of the 48hour time frame they were sampled as retrospective cases with genetic testing only.

**Supplementary Figure 1: Consort diagram of AE-ACEI cases**


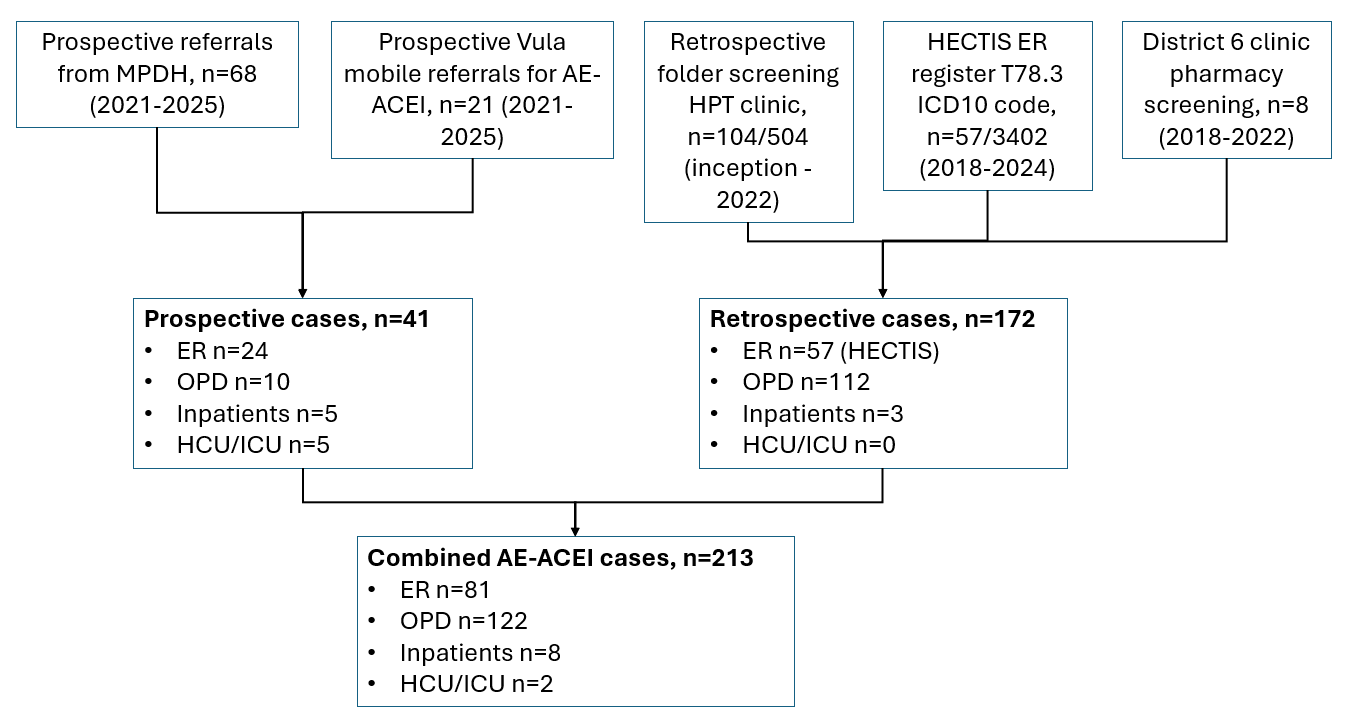


*Vula mobile is the cellular phone application that allows secure referral of patients from clinician to clinician. HECTIS is the electronic platform in the Western Cape for the management of Emergency Centre triage and admissions*

*ER: Emergency Room; HCU: High care unit; ICU: Intensive Care Unit; OPD: Outpatient Department*

**Supplementary Figure 2: Consort diagram of ACEI tolerant controls**


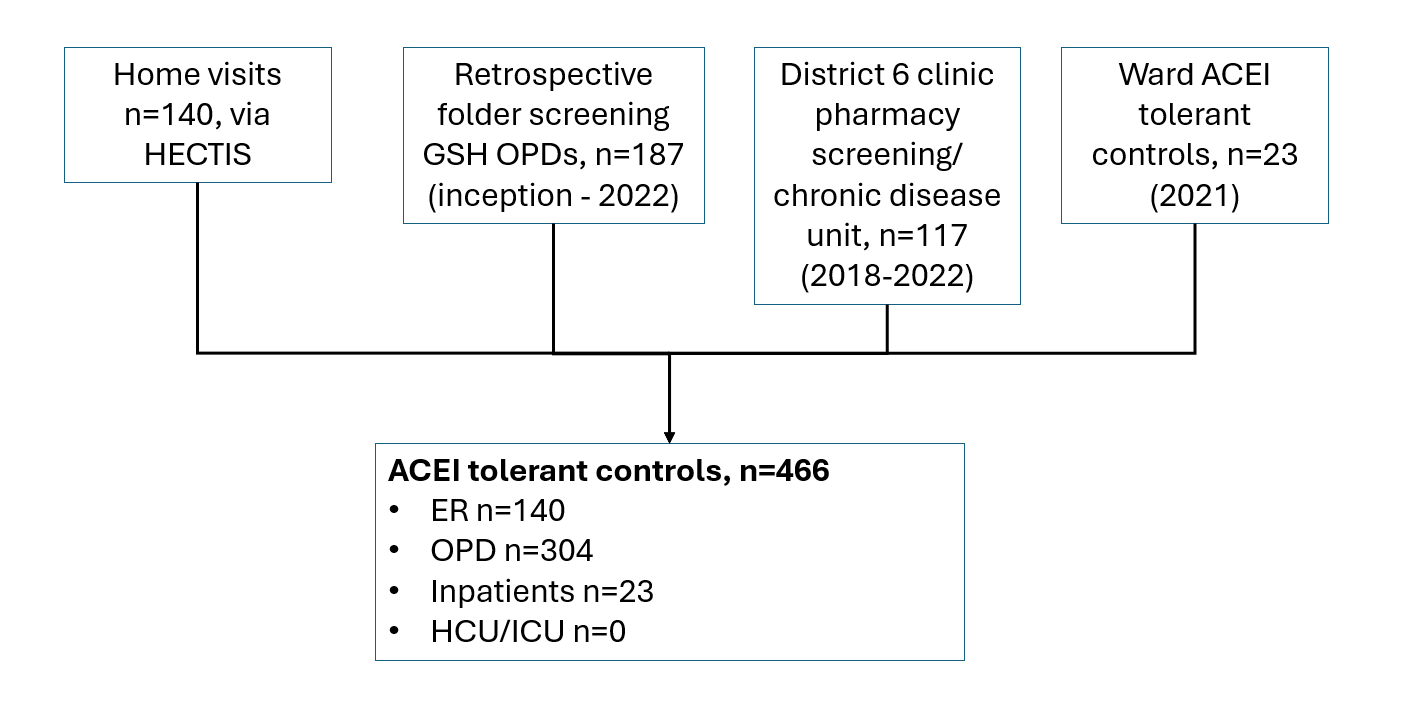


*ER: Emergency Room; HCU: High care unit; ICU: Intensive Care Unit; OPD: Outpatient Department*

**Supplementary Figure 3: PCA plot from previous GWAS**


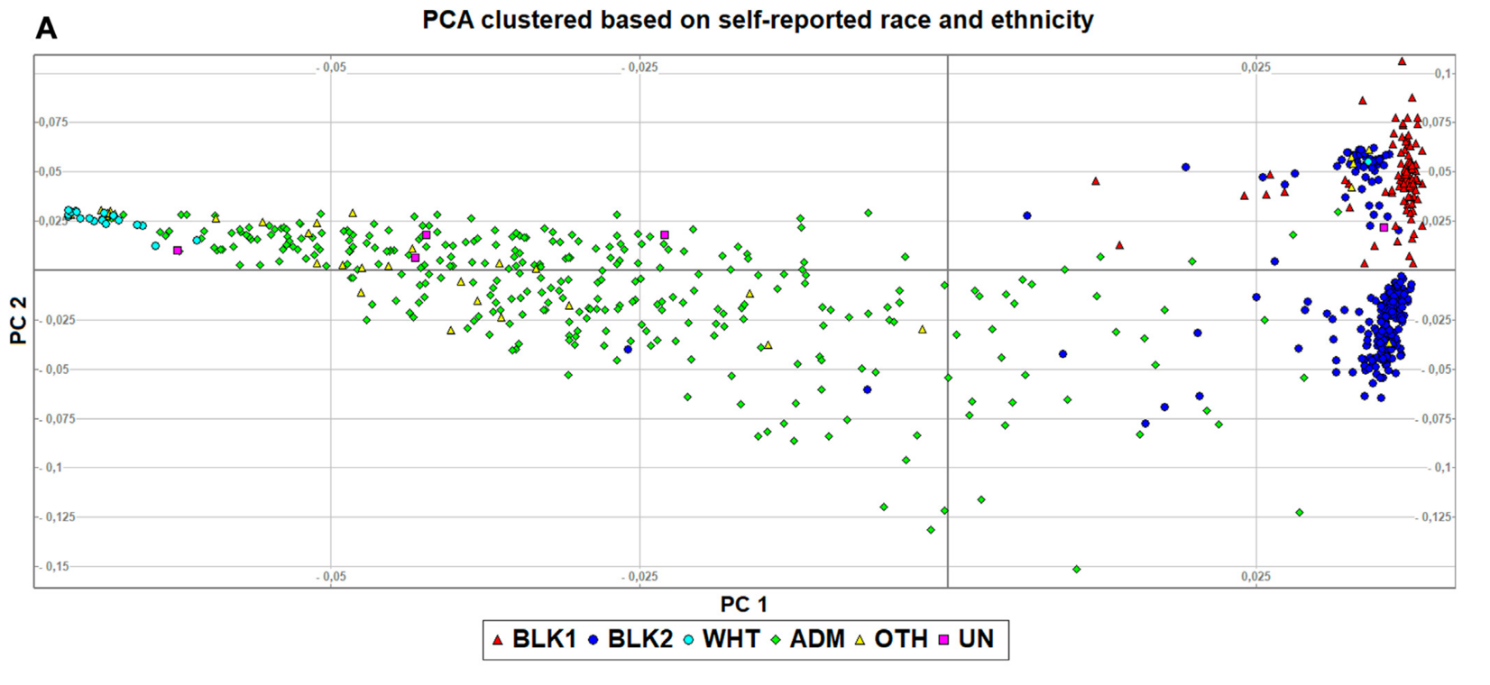


*Adm: admixed ancestry; Blk: black ancestry; Oth: other ancestry; Un: unknown ancestry*

**Supplementary Table 2: Variance Inflation Factor for AE-ACEI Multivariate Model**

| Covariate | VIF | VIF 95% CI | Increased SE | Tolerance | Tolerance 95% CI |
| --- | --- | --- | --- | --- | --- |
| Age | 1.37 | 1.25 – 1.54 | 1.17 | 0.73 | 0.65 – 0.80 |
| Gender | 1.05 | 1.01 – 1.28 | 1.03 | 0.95 | 0.78 – 0.99 |
| Ancestry | 1.41 | 1.29 – 1.59 | 1.19 | 0.71 | 0.63 – 0.78 |
| HIV | 1.18 | 1.10 – 1.33 | 1.09 | 0.85 | 0.75 – 0.91 |
| Atopy | 1.03 | 1.00 – 1.48 | 1.02 | 0.97 | 0.67 – 1.00 |
| Hypertension | 1.17 | 1.09 – 1.32 | 1.08 | 0.86 | 0.76 – 0.92 |
| Elevated cholesterol | 2.31 | 2.05 – 2.64 | 1.52 | 0.43 | 0.38 – 0.49 |
| Cardiac failure | 1.17 | 1.09 – 1.33 | 1.08 | 0.85 | 0.75 – 0.92 |
| Chronic kidney disease | 1.05 | 1.01 – 1.32 | 1.02 | 0.96 | 0.76 – 0.99 |
| Immunosuppression | 1.14 | 1.07 – 1.3 | 1.07 | 0.87 | 0.77 – 0.93 |
| NSAID use | 1.28 | 1.18 – 1-44 | 1.13 | 0.78 | 0.69 – 0.85 |
| Statin use | 2.23 | 1.98 – 2.54 | 1.49 | 0.45 | 0.39 – 0.51 |
| Calcium channel blocker use | 1.03 | 1.00 – 1.51 | 1.02 | 0.97 | 0.66 – 1.00 |
| Hospitalised | 1.12 | 1.05 – 1.28 | 1.06 | 0.89 | 0.78 – 0.95 |

*NSAID: non-steroidal anti-inflammatory drug; VIF: Variance inflation factor*

**Supplementary Table 3 Univariate and Multivariate analysis of risk factors for developing ACEI angioedema within 30 days of initiation of ACEI**

|  | **Univariate** | | | **Multivariate** | | |
| --- | --- | --- | --- | --- | --- | --- |
| **Characteristic** | **OR** | **95% CI OR** | **p-value** | **aOR** | **95% CI aOR** | **p-value** |
| Age | 1.00 | 0.97 – 1.02 | 0.7 | 1.00 | 0.97 – 1.03 | >0.9 |
| Male | 1.54 | 0.85 – 2.78 | 0.2 | 1.69 | 0.83 – 3.48 | 0.2 |
| Black Ancestry | 1.12 | 0.57 – 2.19 | 0.7 | 0.93 | 0.40 – 2.13 | 0.9 |
| Atopy | 1.16 | 0.62 – 2.14 | 0.6 | 1.15 | 0.53 – 2.45 | 0.7 |
| HIV | 1.49 | 0.7 – 3.12 | 0.3 | 1.31 | 0.48 – 3.50 | 0.6 |
| Hypertension | 0.22 | 0.01 – 2.38 | 0.2 | 0.36 | 0.01 – 5.02 | 0.5 |
| Elevated cholesterol | 0.51 | 0.27 – 0.93 | 0.033 | 0.90 | 0.31 – 2.66 | 0.8 |
| Cardiac Failure | 0.70 | 0.22 | 0.50 | 1.05 | 0.27 – 3.74 | >0.9 |
| Chronic Kidney Disease | 0.93 | 0.2 – 3.47 | >0.9 | 0.00 | nil | >0.9 |
| Immunosuppressive drug use | 0.72 | 0.10 – 3.21 | 0.7 | 1.47 | 0.14 – 11.7 | 0.7 |
| NSAID use | 0.6 | 0.26 – 1.27 | 0.20 | 0.96 | 0.33 – 2.63 | >0.9 |
| Statin use | 0.44 | 0.23 – 0.81 | 0.01 | 0.60 | 0.20 – 1.77 | 0.4 |
| Calcium channel blocker use | 0.87 | 0.48 – 1.59 | 0.7 | 0.69 | 0.32 – 1.48 | 0.3 |
| Hospitalised | 1.13 | 0.63 – 2.02 | 0.7 | 1.00 | 0.47 – 2.11 | >0.9 |

**Supplementary Table 4: Atopic conditions and management**

| **Variable** | **All, n=679** | **ACE-I angioedema cases, n=213** | **Controls, n=466** | **P values** |  |
| --- | --- | --- | --- | --- | --- |
| Presence of Atopic disease | 198 (29.2) | 69 (32.3) | 129 (27.6) | 0.201 |  |
| Allergic rhinitis | 117 (59.0) | 37 (53.6) | 80 (62.0) | 0.948 |  |
| Asthma | 56 (28.3) | 29 (42.0) | 27 (20.9) | **0.001** |  |
| Atopic dermatitis | 48 (24.2) | 8 (11.6) | 40 (31.0) | **0.023** |  |
| Previous urticaria | 19 (9.6) | 6 (8.7) | 13 (2.8) | 0.984 |  |
| Allergic rhinitis triggers, n=117 | | | | | |
| House dust mites | 69 (59.0) | 18 (48.6) | 51 (63.8) | 0.318 |  |
| Pollen / spores | 72 (61.5) | 19 (51.4) | 53 (64.0) | 0.335 |  |
| Animal | 22 (18.8) | 6 (16.2) | 16 (20.0) | 0.674 |  |
| Smoke | 28 (23.9) | 9 (24.3) | 19 (23.8) | 0.928 |  |
| Nil known | 7 (6.0) | 1 (2.7) | 6 (7.5) | 0.300 |  |
| Seasonal | 78 (66.7) | 18 (48.6) | 60 (66.7) | **<0.001** |  |
| Perennial | 25 (21.4) | 7 (18.9) | 18 (22.5) | **<0.001** |  |
| Spring | 53 (45.3) | 15 (40.5) | 38 (47.5) | 0.616 |  |
| Winter | 11 (9.4) | 1 (2.7) | 10 (12.5) | 0.094 |  |
| Autumn | 6 (5.1) | 2 (5.4) | 4 (5.0) | 0.610 |  |
| Summer | 20 (17.1) | 4 (10.8) | 16 (20.0) | **<0.001** |  |
| Allergy management | n=198 | n=69 | n=129 |  |  |
| Oral Antihistamines | 68 (34.3) | 19 (27.5) | 49 (38.0) | 0.900 |  |
| Intranasal corticosteroids | 44 (22.2) | 12 (17.4) | 32 (24.8) | 0.989 |  |
| Intranasal saline | 1 (0.5) | 0 (0.0) | 1 (0.7) | 0.730 |  |
| SABA | 43 (31.7) | 20 (29.0) | 23 (17.8) | **0.004** |  |
| LABA | 7 (3.5) | 4 (5.8) | 3 (2.3) | 0.089 |  |
| Inhaled corticosteroids | 27 (13.6) | 11 (15.9) | 16 (12.4) | 0.082 |  |
| Oral corticosteroids | 7 (3.5) | 4 (5.8) | 3 (2.3) | 0.089 |  |

*ACE-I: Angiotensin converting enzyme inhibitor; AR: allergic rhinitis; CKD: chronic kidney disease; HIV: Human immunodeficiency virus; IQR: interquartile range, LABA: long acting beta agonist , SABA: short acting beta agonist*

**Supplementary Table 5: Breakdown of reported race of AE-ACEI cases in literature**

| **Paper** | **Location** | **Number of participants** | **Racial breakdown of AE-ACEI n, (%)^1^** | |
| --- | --- | --- | --- | --- |
| Kostis et al. 2005(1)  RCT | 12 countries:  United States, Canada, the United Kingdom, Spain, Italy, Germany, Belgium, The Netherlands, Poland, Russia, Israel, and Australia | AE-ACEI cases = 86 (0.68%)  Overall cohort n= 12634 | White | 61 (70.9) |
|  |  |  | **Black** | **20 (23.3)** |
|  |  |  | Asian / Pacific | 3 (3.5) |
|  |  |  | Other | 2 (2.3) |
| Kamil RJ et al. 2016(2)  Retrospective review | United States | Cases n=1247  Healthy controls= 6500 | White | 104 (8.34) |
|  |  |  | **African American** | **690 (55.3)** |
|  |  |  | Asian | 9 (0.72) |
|  |  |  | Pacific islander | 5 (0.4) |
|  |  |  | American Indian/Alaskan Native | 3 (0.24) |
|  |  |  | Multiracial | 303 (24.3) |
|  |  |  | Unknown | 133 (10.7) |
|  |  |  | Other |  |
| Chan NJ et al. 2015(3)  Retrospective review | United States | AE-ACEI Cases n=88  Unknown number of folders screened | **African American** | **80 (90.9)** |
|  |  |  | Hispanic | 6 (6.8) |
|  |  |  | Caucasian | 2 (2.3) |
| Brown NJ et al. 1996(4)  Retrospective review | United States | AE-ACEI Cases n=82  Total cohort n= 27834 | **African American** | **53 (64.6)** |
|  |  |  | White | 29 (35.4) |
| Banerji A et al. 2008(5)  Retrospective review | United States (5 sites) | AE-ACEI Cases n=175  Total cohort n=586 AE cases | White | 80 (46) |
|  |  |  | **African American** | **73 (42)** |
|  |  |  | Hispanic | 16 (9) |
|  |  |  | Asian | 2 (1) |
| McDowell et al.  2006(6)  Systematic review and meta-analysis, studies n= 564, studies on ACEI n=5 | United States  *Burkhart 1996(7)*  *Julius 2004(8)*  *Kostis 2004(9)*  *Morimoto 2004(10)^2^*  *Wright 2005(11)* | AE-ACEI Cases  n=470  Overall cohort n=189216 | **Black** | **236 (50.2)** |
|  |  |  | Non black | 234 (49.7) |
| Reichman ME et al. 2017(12) | United States | AE-ACEI Cases  n=6181 | White | 3794 (61.4) |
|  |  |  | Black | 2107 (34.1) |
|  |  |  | Asian | 62 (1.0) |
|  |  |  | Hispanic | 131 (2.12) |
|  |  |  | Other | 87 (1.41) |
| Julius S et al. 2004(8)  Open label clinical trial | United States | AE-ACEI Cases n=39  Safety cohort n= 11404 | **African American** | **13 (33.3)** |
|  |  |  | White | 24 (61.5) |
|  |  |  | Hispanic | 2 (0.5) |
| Wright JT et al. 2005(11)  Prespecified subgroup analysis of ALLHAT, a randomized, double-blind, active-controlled, clinical outcome trial | United States  Canada | Lisinopril AE Cases n=41  Lisinopril cohort n=3794 | **Black** | **23 (56.1)** |
|  |  |  | Non black | 18 (43.9) |
| Bluestein HM et al. 2009(13) | United States | AE-ACEI Cases n=50  (All AE Cases n=166) | White | 104 (63) |
|  |  |  | **Black** | **62 (37)** |
| Chiu AG et al. 2001(14) | United States | AE-ACEI Cases n=74  (All AE Cases n=108) | White | 3 (3.9) |
|  |  |  | **African American** | **71 (95.9)** |
| **Total** | **11/11 studies in United States** | **AE-ACEI Cases n=8533** | **African American / Black AE-ACEI cases** | **3428 (40.2)** |

1. All labels extracted directly from manuscripts

Information not available for the following studies after contacting authors:

- Morimoto T et al. 2003(10)
- Garcia-Saucedo JC, 2021(15)

**Supplementary Table 6: Missingness values for selected covariates**

| **Characteristic, n=679** | **Missing values** | **Percentage, %** |
| --- | --- | --- |
| Ancestry | 118 | 17.4 |
| Fitz Patrick Skin Tone | 16 | 2.4 |
| HIV | 6 | 0.8 |
| Elevated cholesterol | 6 | 0.8 |
| Cardiac disease | 4 | 0.6 |
| Male | 1 | 0.1 |
| Atopy | 1 | 0.1 |
| Hypertension | 0 | 0.0 |
| Cardiac Failure | 1 | 0.1 |
| Chronic Kidney Disease | 0 | 0.0 |
| Immunosuppressive drug use | 0 | 0.0 |
| NSAID use | 0 | 0.0 |
| Statin use | 0 | 0.0 |
| Calcium channel blocker use | 0 | 0.0 |
| Hospitalised | 0 | 0.0 |

**Supplementary Table 7: STROBE Statement—Checklist of items that should be included in reports of** ***case-control studies***

|  | Item No | Recommendation |  |
| --- | --- | --- | --- |
| **Title and abstract** | 1 | (*a*) Indicate the study’s design with a commonly used term in the title or the abstract | ✓ |
|  |  | (*b*) Provide in the abstract an informative and balanced summary of what was done and what was found | ✓ |
| Introduction | | |  |
| Background/rationale | 2 | Explain the scientific background and rationale for the investigation being reported | ✓ |
| Objectives | 3 | State specific objectives, including any prespecified hypotheses | ✓ |
| Methods | | |  |
| Study design | 4 | Present key elements of study design early in the paper | ✓ |
| Setting | 5 | Describe the setting, locations, and relevant dates, including periods of recruitment, exposure, follow-up, and data collection | ✓ |
| Participants | 6 | (*a*) Give the eligibility criteria, and the sources and methods of case ascertainment and control selection. Give the rationale for the choice of cases and controls | ✓ |
|  |  | (*b*) For matched studies, give matching criteria and the number of controls per case | ✓ |
| Variables | 7 | Clearly define all outcomes, exposures, predictors, potential confounders, and effect modifiers. Give diagnostic criteria, if applicable | ✓ |
| Data sources/ measurement | 8* | For each variable of interest, give sources of data and details of methods of assessment (measurement). Describe comparability of assessment methods if there is more than one group | ✓ |
| Bias | 9 | Describe any efforts to address potential sources of bias | ✓ |
| Study size | 10 | Explain how the study size was arrived at | ✓ |
| Quantitative variables | 11 | Explain how quantitative variables were handled in the analyses. If applicable, describe which groupings were chosen and why | ✓ |
| Statistical methods | 12 | (*a*) Describe all statistical methods, including those used to control for confounding | ✓ |
|  |  | (*b*) Describe any methods used to examine subgroups and interactions | ✓ |
|  |  | (*c*) Explain how missing data were addressed | ✓ |
|  |  | (*d*) If applicable, explain how matching of cases and controls was addressed | ✓ |
|  |  | (*e*) Describe any sensitivity analyses | ✓ |
| Results | | |  |
| Participants | 13* | (a) Report numbers of individuals at each stage of study—eg numbers potentially eligible, examined for eligibility, confirmed eligible, included in the study, completing follow-up, and analysed | ✓ |
|  |  | (b) Give reasons for non-participation at each stage | ✓ |
|  |  | (c) Consider use of a flow diagram | ✓ |
| Descriptive data | 14* | (a) Give characteristics of study participants (eg demographic, clinical, social) and information on exposures and potential confounders | ✓ |
|  |  | (b) Indicate number of participants with missing data for each variable of interest | ✓ |
| Outcome data | 15* | Report numbers in each exposure category, or summary measures of exposure | ✓ |
| Main results | 16 | (*a*) Give unadjusted estimates and, if applicable, confounder-adjusted estimates and their precision (eg, 95% confidence interval). Make clear which confounders were adjusted for and why they were included | ✓ |
|  |  | (*b*) Report category boundaries when continuous variables were categorized | ✓ |
|  |  | (*c*) If relevant, consider translating estimates of relative risk into absolute risk for a meaningful time period | ✓ |

**Supplementary Figure 3: Consort diagram of AE-ACEI cases**


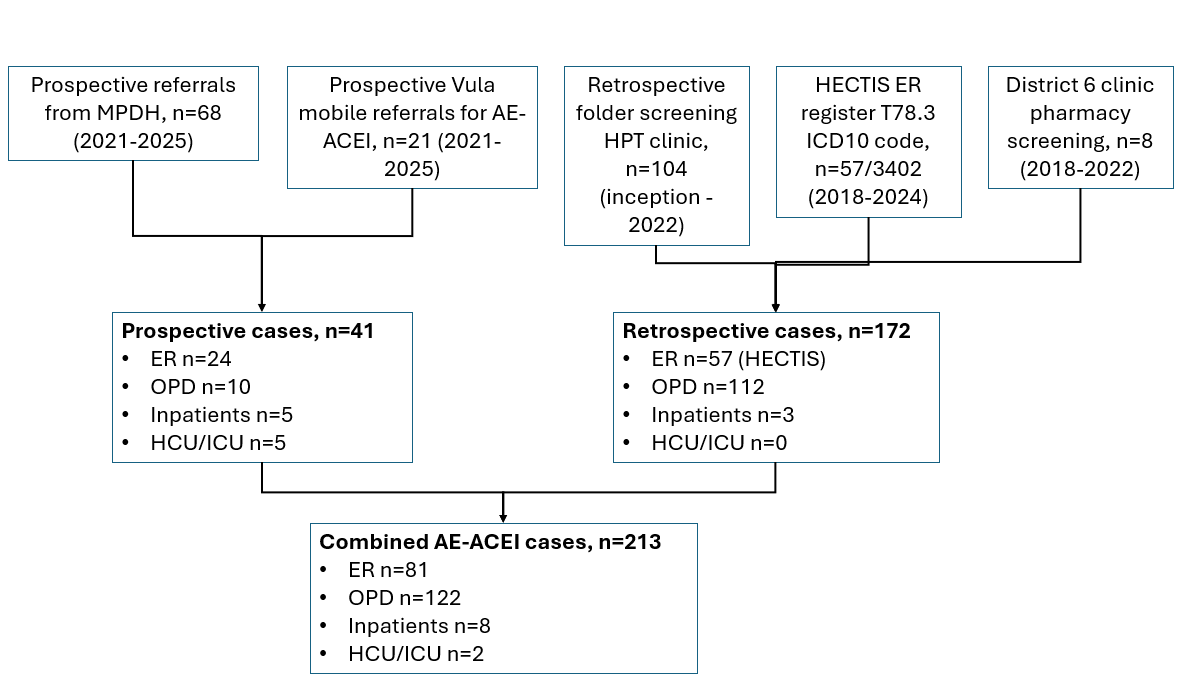


**Supplementary Figure 4: Consort diagram of ACEI tolerant controls**


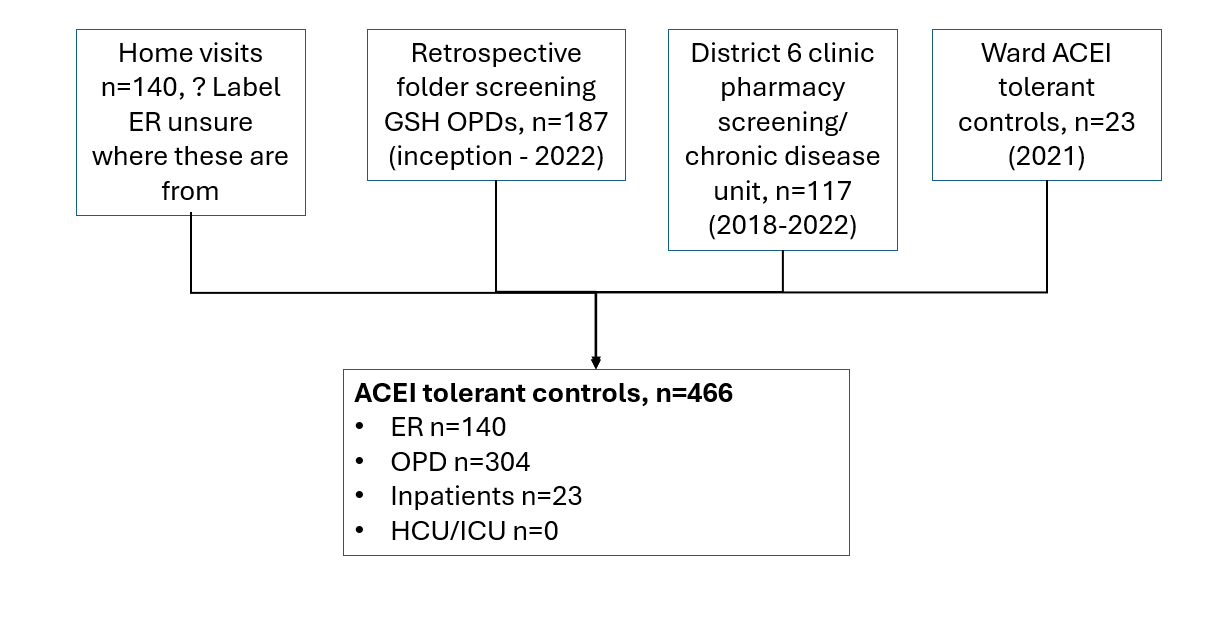


**References**

1. Kostis JB, Kim HJ, Rusnak J, Casale T, Kaplan A, Corren J, et al. Incidence and characteristics of angioedema associated with enalapril. Archives of internal medicine. 2005;165(14):1637-42.

2. Kamil RJ, Jerschow E, Loftus PA, Tan M, Fried MP, Smith RV, et al. Case-control study evaluating competing risk factors for angioedema in a high-risk population. Laryngoscope. 2016;126(8):1823-30.

3. Chan NJ, Soliman AM. Angiotensin converting enzyme inhibitor-related angioedema: onset, presentation, and management. Ann Otol Rhinol Laryngol. 2015;124(2):89-96.

4. Brown NJ, Ray WA, Snowden M, Griffin MR. Black Americans have an increased rate of angiotensin converting enzyme inhibitor‐associated angioedema. Clinical Pharmacology & Therapeutics. 1996;60(1):8-13.

5. Banerji A, Clark S, Blanda M, LoVecchio F, Snyder B, Camargo CA, Jr. Multicenter study of patients with angiotensin-converting enzyme inhibitor-induced angioedema who present to the emergency department. Ann Allergy Asthma Immunol. 2008;100(4):327-32.

6. McDowell SE, Coleman JJ, Ferner RE. Systematic review and meta-analysis of ethnic differences in risks of adverse reactions to drugs used in cardiovascular medicine. Bmj. 2006;332(7551):1177-81.

7. Burkhart DG, Brown NJ, Griffin MR, Ray WA, Hammerstrom T, Weiss S. Angiotensin converting enzyme inhibitor-associated angioedema: higher risk in blacks than whites. Pharmacoepidemiol Drug Saf. 1996;5(3):149-54.

8. Julius S, Cohn JN, Neutel J, Weber M, Turlapaty P, Shen Y, et al. Antihypertensive utility of perindopril in a large, general practice-based clinical trial. J Clin Hypertens (Greenwich). 2004;6(1):10-7.

9. Kostis JB, Packer M, Black HR, Schmieder R, Henry D, Levy E. Omapatrilat and enalapril in patients with hypertension: the Omapatrilat Cardiovascular Treatment vs. Enalapril (OCTAVE) trial. Am J Hypertens. 2004;17(2):103-11.

10. Morimoto T, Gandhi TK, Fiskio JM, Seger AC, So JW, Cook EF, et al. An evaluation of risk factors for adverse drug events associated with angiotensin‐converting enzyme inhibitors. Journal of evaluation in clinical practice. 2004;10(4):499-509.

11. Wright JT, Jr., Dunn JK, Cutler JA, Davis BR, Cushman WC, Ford CE, et al. Outcomes in hypertensive black and nonblack patients treated with chlorthalidone, amlodipine, and lisinopril. Jama. 2005;293(13):1595-608.

12. Reichman ME, Wernecke M, Graham DJ, Liao J, Yap J, Chillarige Y, et al. Antihypertensive drug associated angioedema: effect modification by race/ethnicity. Pharmacoepidemiol Drug Saf. 2017;26(10):1190-6.

13. Bluestein HM, Hoover TA, Banerji AS, Camargo CA, Jr., Reshef A, Herscu P. Angiotensin-converting enzyme inhibitor-induced angioedema in a community hospital emergency department. Ann Allergy Asthma Immunol. 2009;103(6):502-7.

14. Chiu AG, Newkirk KA, Davidson BJ, Burningham AR, Krowiak EJ, Deeb ZE. Angiotensin-converting enzyme inhibitor-induced angioedema: a multicenter review and an algorithm for airway management. Ann Otol Rhinol Laryngol. 2001;110(9):834-40.

15. Garcia-Saucedo JC, Trejo-Gutierrez JF, Volcheck GW, Park MA, Gonzalez-Estrada A. Incidence and risk factors of angiotensin-converting enzyme inhibitor-induced angioedema: A large case-control study. Ann Allergy Asthma Immunol. 2021;127(5):591-2.
